# Effects of Digital Intelligent Interventions on Self-Management of Patients With Diabetic Foot: Systematic Review

**DOI:** 10.2196/64400

**Published:** 2025-03-25

**Authors:** Jinyan Zhou, Shanni Ding, Yihong Xu, Hongying Pan

**Affiliations:** 1 Nursing Department Sir Run Run Shaw Hospital Zhejiang University School of Medicine Hangzhou China

**Keywords:** diabetic foot, self-management, digital intelligent intervention, systematic review, mobile phone

## Abstract

**Background:**

Diabetic foot (DF) is one of the most common and serious complications of diabetes. Effective self-management by patients can delay disease progression and improve quality of life. Digital intelligent technologies have emerged as advantageous in assisting patients with chronic diseases in self-management. However, the impact of digital intelligent technologies on self-management of patients with DF remains unclear.

**Objective:**

This systematic review aimed to determine the effects of digital intelligent interventions on self-management in patients with DF.

**Methods:**

A systematic literature search was conducted across PubMed, Web of Science, Embase, Cumulative Index to Nursing and Allied Health Literature, PsycINFO, Cochrane Central Register of Controlled Trial, ProQuest, China National Knowledge Internet, WanFang, China Science and Technology Journal Database, and SinoMed up to February 6, 2025, to identify eligible articles. Randomized controlled trials (RCTs) that assessed the effects of digital intelligent interventions on self-management of patients with DF were included. In total, 2 researchers independently conducted literature screening, quality assessment, and data extraction. The Cochrane Risk of Bias 2.0 tool (revised version 2019) for RCTs was used to assess the quality of the studies. A qualitative synthesis was conducted on the extracted data.

**Results:**

In total, 1079 articles were retrieved, and 18 RCTs were included. All studies were rated as having a high risk of bias. The digital intelligent interventions in the included studies varied in forms, components, and durations. The intervention forms included WeChat (Tencent Holdings Limited; 7/18, 39%), apps (4/18, 22%), electronic platforms (3/18, 17%), mixed interventions (3/18, 17%), and smartphone thermography (1/18, 6%). The intervention components included self-management education (17/18, 94%), blood glucose and foot condition monitoring (8/18, 44%), self-management supervision and follow-up (6/18, 33%), and other components like foot risk assessment, foot care reminders, visit reminders, and remote consultations. Intervention durations ranged from 5 weeks to 12 months, with the majority (10/18, 56%) lasting 6 months. Among the 18 included studies, 17 studies (17/18, 94%) indicated that, compared with routine care, digital intelligent interventions significantly improved the self-management behaviors of patients with DF, including diabetes control, foot care behaviors, and blood glucose monitoring. Only 1 study (1/18, 6%) showed that the effects of digital intelligent interventions were not significantly different from those of routine care.

**Conclusions:**

In this systematic review, evidence suggests that digital intelligent interventions can improve self-management behaviors and capabilities in patients with DF. However, due to the overall low quality of the included studies, current evidence should be interpreted and applied with caution. This field is still in the exploratory stage, with significant heterogeneity among different studies and a lack of consensus on intervention strategies, necessitating further exploration tailored to different populations. Future RCTs with large sample sizes and rigorous design are needed to develop high-quality evidence.

**Trial Registration:**

PROSPERO CRD42024524473; https://www.crd.york.ac.uk/PROSPERO/view/CRD42024524473

## Introduction

Diabetic foot (DF) refers to foot conditions in patients with diagnosed or past diabetes mellitus (DM), manifesting as one or more of the following: peripheral neuropathy, peripheral arterial disease, infection, ulcers, Charcot neuroarthropathy, gangrene, or amputation [1]. DF is one of the most common and severe chronic complications of DM. Data from the International Working Group on the Diabetic Foot (IWGDF) indicated that 85% of patients with DF were at risk of amputation, with a mortality rate of 50% to 68% within 5 years post amputation, and a 10-year survival rate of only 24% [2]. Compared with patients with typical DM, those with DF face higher treatment costs, more frequent clinic visits, longer treatment journeys, greater physical trauma and psychological burdens, and higher mortality rates [2]. The treatment journey for DF is lengthy, as the disease persists and presents both long-term and intricate aspects, requiring patients to engage in prolonged, continuous, and steadfast self-management. In addition to regular DM self-management activities such as monitoring blood sugar, maintaining a balanced diet, and exercising, the self-management for patients with DF also includes foot monitoring and care such as daily inspection of both feet (temperature, blisters, wounds, etc), wearing well-fitting shoes, wearing seamless socks and changing them daily, washing feet daily and carefully drying them, avoiding the use of heating pads or warmers for the feet, using moisturizers for skin care, but not between the toes, and trimming nails in a straight line [3]. Studies showed that proficient self-management notably enhanced treatment adherence and slowed disease progression in patients with DF, thereby diminishing the likelihood of adverse outcomes like amputations [4]. However, due to factors such as lack of knowledge, inadequate social support, and low self-efficacy, the self-management status of patients with DF is often poor [5].

For self-management of patients with DF, traditional intervention methods often involve health education during hospitalization, followed by follow-ups through phone calls or home visits after discharge, which are time-consuming and costly, and have lower communication efficiency [6]. Digital intelligence is the integration of digitalization and intelligence, including core technology clusters such as big data, cloud computing, artificial intelligence, the internet, the Internet of Things, and 5G [7]. Digital intelligent interventions are developed based on digital intelligence, using technologies driven by digital intelligence, such as mobile apps, machine learning algorithms, virtual reality, wearable devices, and brain-computer interfaces, to provide patients with more convenient and real-time health management solutions, characterized by real-time capability, intelligence, quantifiability, visualizability, and optimizability [8]. Compared with traditional intervention methods, digital intelligent interventions can overcome the limitations of time and space, providing more efficient and personalized self-management support for patients through text, pictures, audio, and video anytime and anywhere [8]. In light of the current poor self-management levels of patients with DF, digital intelligent interventions may offer a potentially promising path forward.

At present, the applications of digital intelligent interventions in the self-management of patients with DF mainly include health monitoring, health education, and follow-up, with forms such as apps, electronic platforms, and wearable devices [9-12]. These technologies provide convenient guidance and support for health care professionals to assist patients in self-management, showing certain advantages in enhancing patient satisfaction. However, applying digital intelligent technologies in the self-management of patients with DF is still in the exploratory stage, with significant differences in intervention forms, intervention durations, and specific strategies among various studies [9-12]. Furthermore, due to methodological challenges such as small sample sizes and insufficient representativeness of the study population [9,10], the effectiveness of these intervention measures in the self-management of patients with DF remains unclear. To address this knowledge gap, there is an urgent need for a systematic review of digital intelligent interventions’ impact on self-management among patients with DF. To the best of our knowledge, this is the first systematic review focusing on the impact of digital intelligent interventions on self-management in patients with DF. Such a study would help clarify the role of digital intelligent technologies in DF management and provide valuable insights for improving self-management strategies tailored to the unique needs of patients with DF.

## Methods

### Overview

A systematic review was carried out following the guidelines outlined in the Cochrane Handbook for Systematic Reviews of Interventions, and the reporting standards specified in the PRISMA (Preferred Reporting Items for Systematic Reviews and Meta-Analyses) Statement 2020 [13]. Based on the registered protocol, we had initially intended to use RevMan 5.3 software (Cochrane) for data synthesis and meta-analysis. However, due to the great heterogeneity of the included studies and the limited availability of comparable data, a quantitative analysis could not be performed. As a result, we opted for a qualitative analysis to summarize the findings. This deviation from the original protocol is reported here to maintain transparency.

### Eligibility Criteria

Eligibility criteria were meticulously devised by the PICOS (P: population, I: intervention, C: comparison, O: outcome, S: study design) framework [13]. This framework entails defining the Population (P), Intervention (I), Comparison (C), Outcome (O), and Study design (S) characteristics to ensure clarity and consistency in study selection.

### Population

Patients with a confirmed diagnosis of DF. This study used the definition of DF according to the IWGDF [1], which refers to a foot condition in individuals with current or previously diagnosed DM characterized by one or more of the following: peripheral neuropathy, peripheral artery disease, infection, ulcer, neuro-osteoarthropathy, gangrene, or amputation.

### Intervention

Digital intelligent interventions. As mentioned in the introduction, digital intelligent interventions refer to health management solutions that integrate both digital technologies and intelligent features. These interventions rely not only on digital tools but also on intelligent capabilities, enabling real-time, dynamic, and personalized health management through data analysis, real-time feedback, and personalized adjustments. Intervention forms include apps, electronic platforms, wearable devices, and so on. It is important to emphasize that simple digital tools, such as static and noninteractive health education videos, although possessing certain digital characteristics, do not fall within the scope of digital intelligent interventions due to the lack of intelligent analysis and personalized adjustments. Furthermore, the digital intelligent intervention was the core intervention of the intervention group.

### Comparison

Nondigital intelligent interventions refer to health management approaches that either lack digital technology entirely or use digital tools without intelligent features. These interventions include conventional, nondigital methods such as face-to-face health education, printed materials, or standard counseling. They may also involve digital tools that are not equipped with intelligent functionalities, such as static, noninteractive health education videos, which provide information but do not offer real-time feedback, personalized adjustments, or data-driven insights. Essentially, these interventions lack the dynamic, real-time, and personalized capabilities that characterize digital intelligent interventions.

### Outcome

Patients’ self-management behavior or self-management ability. For patients with DF, self-management activities include monitoring blood sugar, a healthy diet, regular exercise, medication adherence, foot monitoring, and care. Commonly used scales to assess the self-management behavior or ability of patients with DF include the Summary of Diabetes Self-Care Activities (SDSCA) [14], Type 2 Diabetes Self-Care Scale (2-DSCS) [15], and Diabetic Foot Self-Care Questionnaire of the University of Malaga (DFSQ-UMA) [16].

### Study Design

This review exclusively included randomized controlled trials (RCTs) with full text that were published in either English or Chinese.

Based on the above PICOS, there were certain inclusion and exclusion criteria for this study (Textbox 1).

**The inclusion and exclusion criteria for screening studies.**

**Inclusion criteria:**

- Population: patients with a confirmed diagnosis of diabetic foot.
- Intervention: the intervention group received digital intelligent interventions.
- Comparison: the control group received nondigital intelligent interventions.
- Outcome: patients’ self-management behavior or self-management ability.
- Study design: randomized controlled trial.

**Exclusion criteria:**

- Duplicate published articles.
- No full text available.
- Not in Chinese or English.

### Search Strategy

A systematic literature search was conducted across PubMed, Web of Science, Embase, Cumulative Index to Nursing and Allied Health Literature, PsycINFO, Cochrane Central Register of Controlled Trials, ProQuest, China National Knowledge Internet, WanFang, China Science and Technology Journal Database, and SinoMed up to February 6, 2025. A combination of subject terms and free words was used with logical operators for searching. In addition, the reference lists of included studies were scrutinized, and when necessary, studies were manually retrieved to identify other potential studies meeting the inclusion criteria. The detailed search strategies for each database are provided in Table S1 in Multimedia Appendix 1. The detailed information on the PRISMA checklist can be found in Multimedia Appendix 2.

### Study Selection

References from databases underwent deduplication using NoteExpress (Beijing Aiqinhai Software Company). In total, 2 reviewers (JZ and SD) independently assessed the titles and abstracts of studies for initial screening. Subsequently, a full-text–level assessment was conducted by 2 independent reviewers (JZ and SD) to select the eligible articles for inclusion. Any disagreements were resolved through discussion or arbitration involving a third researcher (YX).

### Quality Assessment

Two researchers (JZ and SD) independently conducted quality assessments using the Cochrane Risk of Bias 2.0 tool (revised version 2019, developed by Sterne et al [17]) for RCTs, which evaluates five bias domains, that are (1) randomization process, (2) deviations from intended interventions, (3) missing outcome data, (4) measurement of outcome, and (5) selection of reported results. Each domain and the overall bias were assessed as either low risk of bias, some concerns, or high risk of bias [13]. Any disagreements were resolved by seeking input from a third senior expert (YX).

### Data Extraction

The data extraction process adhered to the “checklist of items for data collection form” outlined in the Cochrane Handbook and aligned with the specific PICOS principle of this review. In total, 2 reviewers (JZ and SD) independently extracted the following data from each study: first author, year published, country, included centers, participant, intervention content, intervention duration, use of intention-to-treat (ITT), outcome measures, and results of interest. Any disparities were addressed through discussion, leading to a consensus on all extracted data. In cases where additional data were needed, the authors of the included studies were contacted for further information.

### Data Synthesis

The differences in the intervention methods, intervention durations, outcome assessment methods, and reporting formats across the included studies led to significant heterogeneity among the studies, hindering the quantitative synthesis of the data. Therefore, this study focused on qualitative synthesis. Tables 1 and 2 summarize the basic characteristics of the included studies. Table 3 summarizes the intervention details and the intervention outcomes of the included studies. We narratively synthesized the extracted data in terms of the population of intervention, digital intelligent intervention forms, components and durations, control group interventions, evaluation indicators and tools, and the effects of digital intelligent interventions on the self-management of patients with DF.

**Table 1:** The basic characteristics of the participants in the included studies.

Authors, year	Country	Participant						
		Type^a^	Number (I^b^/C^c^)	Age (years), mean (SD)			Male, n (%)	
				I	C	I		C
Qin et al, 2023 [12]	Indonesia	Diabetic patients with a history of DFU^d^ (Wagner grade 0)	60/60	61.7 (10.78)	59.7 (10.61)	29 (48)		29 (48)
Marques et al, 2023 [10]	Brazil	T2DM^e^ medical diagnosis and foot at risk (Wagner grade 0)	20/22	—^f^	—	—		—
Firdaus et al, 2023 [9]	Malaysia	Last 2 readings of A1c^g^ more than 6.5, has at least stage 1 or above risk of foot ulcer development (Wagner grade 0)	29/29	50.03 (8.03)	57.59 (7.18)	9 (31)		11 (38)
Xu et al, 2023 [18]	China	Patients with DF^h^ discharged after unilateral transverse tibial bone transport, Wagner grade was 3-4	21/21	64.5 (8.6)	65.8 (8.4)	18 (86)		19 (90)
Zhang, 2022 [6]	China	Patients with DF with Wagner grade 0	46/46	65.69 (3.78)	65.69 (3.78)	25 (54)		26 (57)
Zhang et al, 2021 [19]	China	Patients with DF with Wagner grade 1-2	43/43	64.42 (4.20)	66.31 (4.56)	27 (63)		24 (56)
Chu et al, 2021 [20]	China	Patients with DF	60/60	51.49 (12.73)	52.42 (11.40)	37 (62)		33 (55)
Li et al, 2021 [21]	China	Patients at high risk of DF (Wagner grade 0)	58/58	—	—	31 (53)		35 (60)
Ye and Yu, 2021 [22]	China	Patients with DF	29/29	62.9 (9.4)	63.2 (8.4)	18 (62)		15 (52)
Qu, 2020 [23]	China	Patients with type 2 diabetic foot with Wagner grade 1-4	40/40	64.5 (5.3)	64.0 (4.5)	28 (70)		30 (75)
Xie et al, 2020 [24]	China	Patients with DF with Wagner grade 0	100/95	61.1 (7.3)	61.9 (6.8)	48 (48)		48 (51)
Xu, 2019 [25]	China	Patients with DF	46/46	54.86 (6.07)	53.15 (5.16)	24 (52)		25 (54)
Liu, 2019 [26]	China	Patients with DF (Wagner grade 0-3)	26/32	64.73 (11.85)	65.09 (11.54)	14 (44)		18 (69)
Liu et al, 2018 [27]	China	Patients with DF with Wagner grade 0	90/90	59.8 (9.6)	60.2 (9.3)	62 (62)		64 (64)
Qin et al, 2020 [28]	China	Patients with DF with Wagner grade 0	67/67	53.19 (7.85)	53.84 (8.02)	39 (58)		41 (61)
Hu, 2018 [29]	China	Patients with DF with Wagner grade 0	50/47	56.16 (10.24)	58.34 (8.97)	31 (62)		29 (62)
Bai et al, 2022 [30]	China	Patients with DF with Wagner grade 0-2	67/67	63.94 (9.03)	63.07 (8.52)	41 (61)		37 (55)
Wang et al, 2023 [31]	China	Patients with DF with Wagner grade 0-2	64/64	63.07 (9.12)	62.12 (9.05)	36 (56)		34 (53)

^a^This study applied the Wagner grading system to classify patients with diabetic foot, which is currently the most widely used grading system in clinical practice and research. Grade 0 indicates risk factors for foot ulcers but currently no ulcers are present, while grades 1 and above indicate varying degrees of ulcers or necrosis. A higher grade indicates a greater severity of the disease. The corresponding grade was extracted from the original literature or graded according to its description (the grade in parentheses indicates our grading according to the description), if the original literature had neither grade nor sufficient description to judge, the grade was not recorded.

^b^I: intervention group.

^c^C: control group.

^d^DFU: diabetic foot ulcer.

^e^T2DM: type 2 diabetes mellitus.

^f^Not available.

^g^A_1C_: glycated hemoglobin.

^h^DF: diabetic foot.

**Table 2:** Interventions and results of the included studies.

Authors, year	Digital intelligent intervention, content	Control intervention, content	Results of interest (I^a^ vs C^b^)	*P* value
Qin et al, 2023 [12]	Smartphone thermography evaluation+personalized foot care and education	Usual care and education using a booklet	LSM^c^ 67.5 (SE 1.20) versus LSM 51.9 (SE 1.52)	<.001
Marques et al, 2023 [10]	Standard nursing consultations+application use	Standard nursing consultations	Mean 4.52 (SD 0.78 versus mean 4.51 (SD 0.93)	.99
Firdaus et al, 2023 [9]	Diabetic Care Self-Management Mobile Health Application Program	Standard care	After controlling for age, monthly income, and family history, the foot care behavior of the intervention group was significantly better than that of the control group; *F*_1_=30.374	<.001
Xu et al, 2023 [18]	“Internet+” follow-up nursing intervention (WeChat group, WeChat official account, and blood glucose monitoring app)	Health guidance was given before discharge and routine telephone follow-up	The scores of self-care skills, self-care responsibility, self-concept, and health knowledge in the intervention group were higher than those in the control group	<.01; <.01; .02; .03, respectively
Zhang, 2022 [6]	Transitional care based on WeChat platform (WeChat patient group, WeChat official account, educational knowledge in the form of text, pictures, cartoons, audio, and video)	Telephone follow-up was conducted after discharge	Mean 65.11 (SD 6.15) versus mean 54.26 (SD 7.94)	<.001
Zhang et al, 2021 [19]	Goal-oriented hospital-community linkage continuous nursing care (WeChat official account, WeChat patient group)	Health guidance was given before discharge and regular telephone follow-up after discharge	Mean 58.65 (SD 5.46) versus mean 53.44 (SD 5.81)	<.001
Chu et al, 2021 [20]	WeChat platform follow-up and continuous care (WeChat official account, WeChat patient group)	Routine telephone follow-up and outpatient education	The scores of self-management ability in all dimensions (prevention and treatment of high and low blood glucose, foot care, blood glucose monitoring, reasonable diet, medical compliance, and regular exercise) in the intervention group were higher than those in the control group	<.05
Li et al, 2021 [21]	The diabetic foot app was used for remote management after discharge	Telephone follow-up	Self-monitoring of blood glucose: n=36 (62%) versus n=18 (31%); Regular return visit: n=49 (84%) versus n=33 (57%)	<.05; <.05, respectively
Ye and Yu, 2021 [22]	Transitional care based on WeChat platform (WeChat official account, WeChat patient group)	Routine nursing follow-up	The scores of medication, exercise, diet, blood glucose measurement, and foot care in the intervention group were significantly higher than those in the control group	<.001
Qu, 2020 [23]	Routine nursing+continuing care by WeChat	Routine nursing	The proportion of self-management ability indicators in the intervention group was higher than that in the control group	<.001
Xie et al, 2020 [24]	Internet+blood glucose management (blood glucose management platform+blood glucose manager app)	Conventional blood glucose management	The scores of all dimensions (healthy diet, exercise, self-monitoring of blood glucose, monitoring of blood glucose according to medical advice, self-examination of feet, and medication) in the intervention group were significantly higher than those in the control group	<.001; <.001; <.001; <.001; .01; <.001, respectively
Xu, 2019 [25]	Extended care group combined with “Internet+” mode (WeChat, SMS, QQ [Shenzhen Tencent Computer System Co Ltd] and other internet communication tools, internet extended nursing platform)	Routine nursing	Mean 87.36 (SD 9.27) versus mean 71.68 (SD 9.68)	<.05
Liu, 2019 [26]	Telephone follow-up after discharge+DF app management	Telephone follow-up after discharge	Blood glucose monitoring: n=15 (58%) versus n=10 (31%); Regular follow-up visits: n=16 (62%) versus n=12 (38%); Regular dressing changes: n=22 (85%) versus n=18 (56%)	.04; .07; .02, respectively
Liu et al, 2018 [27]	Transitional care based on WeChat platform (WeChat group, educational knowledge in the form of text, pictures, cartoons, and video)	Telephone follow-up	The scores of all dimensions (reasonable diet, regular exercise, compliance with doctor’s advice, blood glucose monitoring, foot care, prevention of high and low blood glucose) in the intervention group were significantly higher than those in the control group	<.01; <.001; <.001; <.001; <.001; <.001, respectively
Qin et al, 2020 [28]	Transitional care based on WeChat platform (WeChat group, educational knowledge in the form of text, pictures, cartoons, and video)	Routine nursing and telephone follow-up	Mean 74.75 (SD 7.45) versus mean 54.34 (SD 6.13)	<.05
Hu, 2018 [29]	Routine education+telephone follow-up education+platform management	Routine education+telephone follow-up education	Mean 32.19 (SD 4.31) versus mean 28.14 (SD 6.12)	<.001
Bai et al, 2022 [30]	Hospital-community integrated management of hierarchical medical system by medical alliance comanagement	Routine management	The scores of all dimensions (personal care, foot care, and wearing shoes and socks) in the intervention group were significantly higher than those in the control group	<.001; <.001; .001, respectively
Wang et al, 2023 [31]	Medical alliances co-manage hierarchical medical system (Medical alliance information platform, remote consultation)	Routine nursing	The scores of all dimensions (personal care, foot care, and wearing shoes and socks) in the intervention group were significantly higher than those in the control group	.007; .003; .001, respectively

^a^I: intervention group.

^b^C: control group.

^c^LSM: least squares mean.

**Table 3:** Intervention details and outcomes of the included studies.

Authors, year	Participant			Digital intelligent intervention form		Digital intelligent intervention component		Control group intervention		Intervention duration		Evaluation indicator		Evaluation tool		Whether the digital intelligent intervention is effective compared with conventional intervention
	Wagner grade	Number (I^a^/C^b^)														
Qin et al, 2023 [12]	0	60/60	Smartphone thermography		Foot risk assessment		Routine risk assessment		6 m		Foot care behavior		DFCB^c^ questionnaire		Yes	
Marques et al, 2023 [10]	0	20/22	App		Foot care education		Face-to-face education		3 m		Self-care activities with diabetes		QAD^d^		No	
Firdaus et al, 2023 [9]	0	29/29	App		Foot care education, foot care reminders, and foot care behavior supervision		Face-to-face education		5 w		Foot care behavior		DFSBS^e^		Yes	
Xu et al, 2023 [18]	3-4	21/21	WeChat+app		Self-management education, blood glucose monitoring, self-management behavior follow-up		Face-to-face education and telephone follow-up		6 m		General self-management ability		Self-care Agency Scale		Yes	
Zhang, 2022 [6]	0	46/46	WeChat		Self-management education, self-management behavior follow-up		Telephone follow-up and education		—^f^		Diabetes self-management ability		Diabetes Self-management Behavior Scale		Yes	
Zhang et al, 2021 [19]	1-2	43/43	WeChat		Self-management education, self-management behavior follow-up		Face-to-face education and telephone follow-up		6 m		Diabetes self-management ability		SDSCA^g^		Yes	
Chu et al, 2021 [20]	Ungraded	60/60	WeChat		Self-management education		Face-to-face education and telephone follow-up		12 w		Diabetes self-management ability		2-DSCS^h^		Yes	
Li et al, 2021 [21]	0	58/58	App		Foot care education, blood glucose, and foot monitoring		Telephone follow-up		6 m		Specific behaviors		Self-monitoring of blood glucose and regular return visit		Yes	
Ye and Yu, 2021 [22]	Ungraded	29/29	WeChat		Self-management education		Telephone follow-up		6 m		Diabetes self-management ability		2-DSCS		Yes	
Qu, 2020 [23]	1-4	40/40	WeChat		Self-management education, blood glucose monitoring		Face-to-face education		—		Specific behaviors		Listen to a lecture on diabetes, discuss treatment options with the doctor, self-monitoring of blood glucose, remeasuring blood glucose at the hospital, master the method of foot examination, master the method of insulin injection		Yes	
Xie et al, 2020 [24]	0	100/95	Electronic platform+app		Blood glucose monitoring and blood glucose management education		Face-to-face education and telephone follow-up		12 m		Diabetes self-management ability		SDSCA		Yes	
Xu, 2019 [25]	Ungraded	46/46	WeChat+electronic platform		Self-management education, disease monitoring		Face-to-face education using a booklet, telephone follow-up		3 m		Foot care behaviors		Self-management Model Questionnaire		Yes	
Liu, 2019 [26]	0-3	26/32	App		Foot care education, reminders for subsequent visits		Telephone follow-up		3 m		Specific behaviors		Blood glucose monitoring, regular follow-up visits, regular dressing changes		Partly yes	
Liu et al, 2018 [27]	0	90/90	WeChat		Foot care education, blood glucose, and foot monitoring		Telephone follow-up		6 m		Diabetes self-management ability		2-DSCS		Yes	
Qin et al, 2020 [28]	0	67/67	WeChat		Foot care education, blood glucose, and foot monitoring		Face-to-face education and telephone follow-up		6 m		Diabetes self-management ability		Diabetes Self-management Behavior Scale		Yes	
Hu, 2018 [29]	0	50/47	Electronic platform		Foot care education, blood glucose, and foot monitoring		Face-to-face education and telephone follow-up		6 m		Diabetes self-management ability		SDSCA		Yes	
Bai et al, 2022 [30]	0-2	67/67	Electronic platform		Self-management education, self-management behavior follow-up, hospital-community remote consultation and two-way referral		Face-to-face education and telephone follow-up		6 m		Foot care behaviors		DFSQ-UMA^i^		Yes	
Wang et al, 2023 [31]	0-2	64/64	Electronic platform		Self-management education, self-management behavior follow-up, hospital-community remote consultation and two-way referral		Face-to-face education and telephone follow-up		6 m		Foot care behaviors		DFSQ-UMA		Yes	

^a^I: intervention group.

^b^C: control group.

^c^DFCB: diabetic foot care behavior.

^d^QAD: Diabetes Self-care Activities Questionnaire.

^e^DFSBS: Diabetic Foot Selfcare Behavior Scale.

^f^Not available.

^g^SDSCA: Summary of Diabetes Self-Care Activities.

^h^2-DSCS: Type 2 Diabetes Self-care Scale.

^i^DFSQ-UMA: diabetic foot self-care questionnaire of the University of Malaga.

## Results

### Search Results and Study Selection

**Figure 1:**
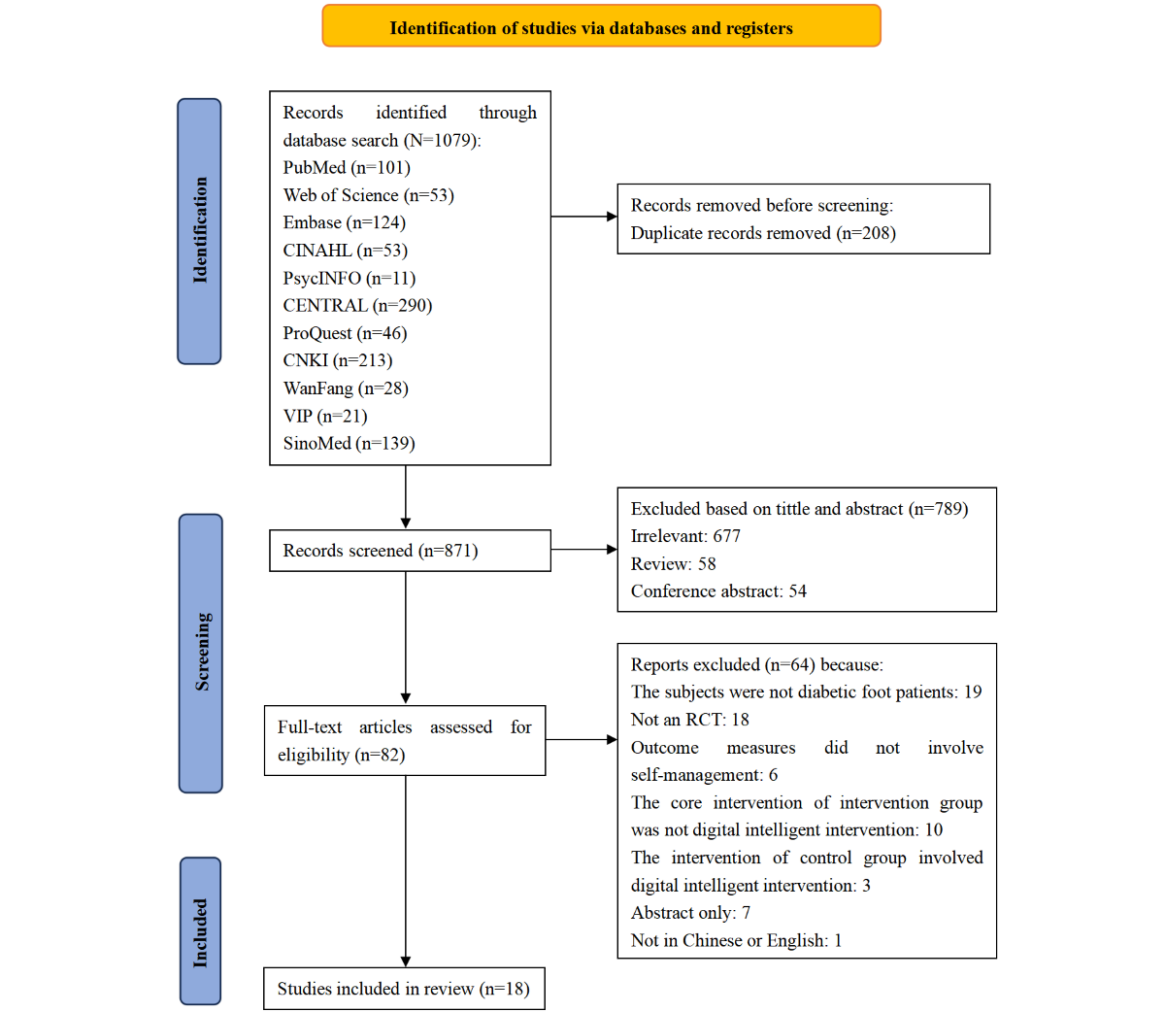
PRISMA flow chart for study selection. RCT: randomized controlled trial.

The flowchart of the literature screening process and reasons for exclusion is illustrated in Figure 1. A total of 1079 records were identified through databases and registration systems. After removing duplicates and screening the titles and abstracts, we conducted a full-text review of 82 articles. Finally, 18 RCTs were included.

### Study Characteristics

Table 1 summarizes the basic characteristics of the participants in the included studies. Table 2 summarizes the interventions and results of the included studies. Details of the included studies are provided in Table S2 in Multimedia Appendix 1. The publication years were from 2018 to 2023 [6,9,10,12,18-31], and 78% (14/18) of the studies were published in the past 5 years (2020 and beyond) [6,9,10,12,18-24,28,30,31]. Except for 1 study [12] conducted at 2 centers, all other studies [6,9,10,18-31] were single-center studies. Studies were conducted in Indonesia [12], Brazil [10], Malaysia [9], and China [6,18-31], with sample sizes ranging from 42 to 195. The age of participants ranged from 50.03 to 66.31, and the proportion of males ranged from 31% to 90%. The intervention forms for the intervention group included WeChat (Tencent Holdings Limited) [6,19,20,22,23,27,28], app [9,10,21,26], electronic platform [29-31], smartphone thermography [12], and mixed interventions (WeChat+app [18], electronic platform+app [24], and WeChat+electronic platform [25]). The control group primarily received standard or usual care interventions focused on diabetes management and foot care. The interventions included health education, health monitoring and follow-up through paper leaflets [12,25], face-to-face communication or examination [9,10,12,18-20,23-25,28-31], and phone calls [6,18-22,24-31]. The intervention duration ranged from 12 months [24] and 6 months [12,18,19,21,22,27-31] to 3 months [10,20,25,26] and 5 weeks [9]. The measurement tools for outcome indicators included Self-care Agency Scale [18], SDSCA [6,19,24,28,29], 2-DSCS [20,22,27], Diabetes Self-care Activities Questionnaire (QAD) [10], Diabetes Self-management Behavior Scale [6,28], DFSQ-UMA [30,31], diabetic foot care behavior (DFCB) [12], Diabetic Foot Selfcare Behavior Scale (DFSBS) [9], and Self-management Model Questionnaire [25]. In addition, some studies [21,23,26] evaluated the self-management behavior of patients by observing their behaviors such as blood glucose monitoring and regular return visits. In terms of the reporting form of the results, some studies [6,10,12,19,25,28,29] compared the total score of the scale between the intervention group and the control group, some [9,18,20,22,24,27,30,31] compared the scores of each dimension of the scale between the 2 groups, and others [21,23,26] compared the number and proportion of people who adopted self-management behaviors between the 2 groups.

### Study Quality

**Figure 2:**
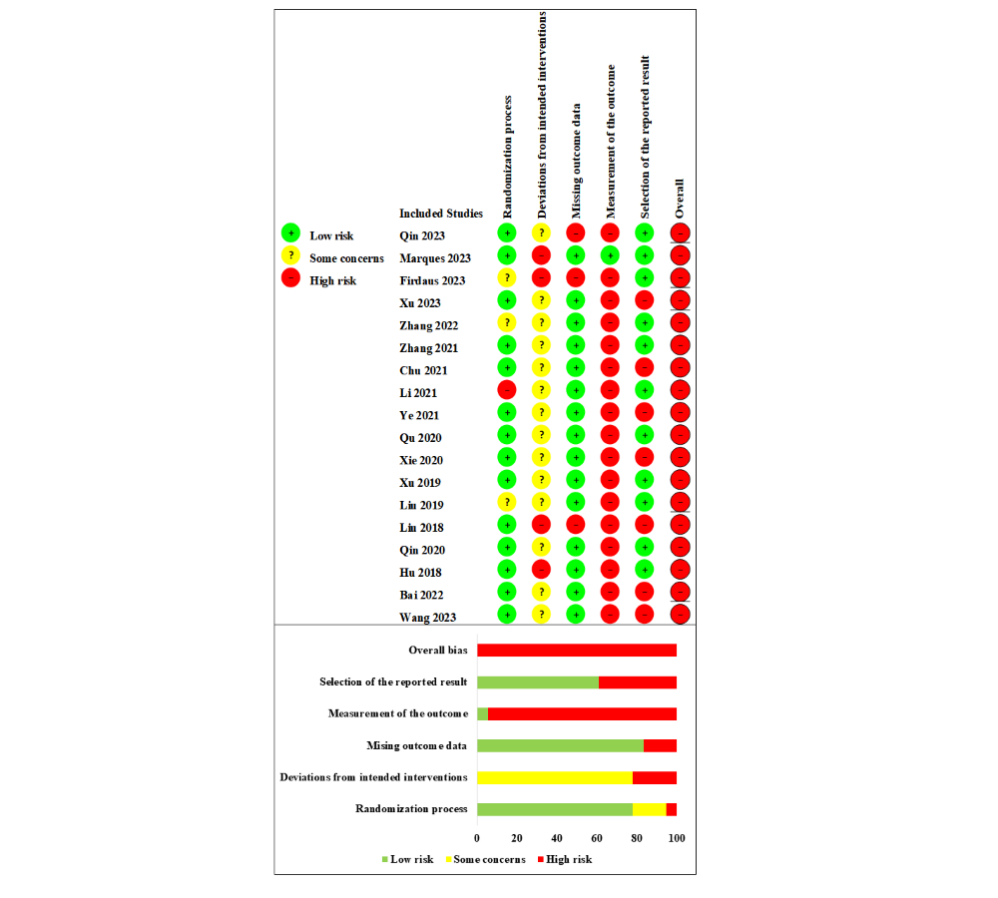
Risk of bias summary: review authors’ judgments about the risk of bias item for each included study [6,9,10,12,18-31].

All of the 18 studies were assessed as having an overall high risk of bias (Figure 2 [6,9,10,12,18-31]). In the randomization process domain, 2 studies [6,26] lacked details on randomization, and the results of baseline between-group comparisons in 1 study [9] suggested potential differences, raising some concerns. One study [21] grouped participants based on odd and even discharge orders, deemed high risk due to inadequate concealment of allocation sequence before grouping. In the domain of deviations from the intended interventions, only 1 study [10] implemented double-blinding, 1 study [29] did not blind participants, 1 study [12] did not blind participants or researchers, and information on blinding was missing in the remaining studies [6,9,18-31]. All studies lacked sufficient information to determine whether intervention deviations from the expected were due to the trial environment. 14 studies [6,12,18-26,28,30,31] conducted ITT analyses, while four studies [9,10,27,29] excluded some randomized participants in their analyses. In the domain of missing outcome data, 5 studies [9,10,12,27,29] had a considerable amount of missing outcome data. The reasons for participant attrition in 2 studies [9,12] were health-related, in 2 studies [10,29] were unrelated to health, and in 1 study [27], the reasons for attrition could not be determined due to insufficient information. In the domain of measurement of the outcome, 3 studies [21,23,26] did not use standardized measurement scales, and 2 studies [6,23] did not specify the measurement timing. In 2 studies [12,29], self-reported scales were used for measurement without blinding participants, so the assessors were aware of the interventions received by the participants. For the 12 studies [6,9,18-20,22,24,25,27,28,30,31], the lack of information made it impossible to determine whether assessors were aware of the interventions received by the participants. Since self-reported scales were used for measurement in the studies [6,9,12,18-20,22,24,25,27-31], which inherently carried subjectivity, knowing the interventions received by the participants could potentially influence the outcomes. In the selection of the reported result domain, 7 studies [18,20,22,24,27,30,31] were rated as high risk due to not reporting the total scale score and total score comparison results between groups.

### Population of Intervention

The intervention details and the intervention outcomes of the included studies are shown in Table 3. The intervention population consisted of patients with DF. In the included studies, most of the participants were classified as Wagner grade 0 [6,9,10,12,21,24,27-29] (9 out of 18 studies, 50%), while some studies included patients with Wagner grade 0-2 [30,31] or Wagner grade 0-3 [26], and others included patients with Wagner grade 1-2 [19], Wagner grade 1-4 [23], or Wagner grade 3-4 [18]. Wagner grade 0 indicates the absence of foot ulcers but the presence of risk factors for ulcer development (such as a history of foot ulcers, peripheral neuropathy, or peripheral arterial disease). Wagner grade 1 and above indicates the presence of foot ulcers or necrosis of varying severity. Some studies [20,22,25] did not apply the Wagner classification system to assess the severity of foot disease in the patients (3 out of 18 studies, 17%).

### Digital Intelligent Intervention Forms, Components, and Durations

Digital intelligent intervention forms, components, and durations of the included studies (N=18) are shown in Multimedia Appendix 3. The forms of digital intelligent interventions included WeChat (7 studies [6,19,20,22,23,27,28], 39%), app (4 studies [9,10,21,26], 22%), electronic platforms (3 studies [29-31], 17%), mixed interventions (WeChat+app [18], WeChat+electronic platform [25], app+electronic platform [24], 3 studies, 17%), and smartphone thermography (1 study [12], 6%). The components of digital intelligent interventions included self-management education (17 studies [6,9,10,18-31], 94%), blood glucose and foot condition monitoring (8 studies [18,21,23-25,27-29], 44%), self-management supervision and follow-up (6 studies [6,9,18,19,30,31], 33%), foot risk assessment (1 study [12], 6%), foot care reminders (1 study [9], 6%), reminders for subsequent visits (1 study [26], 6%), and hospital-community remote consultation and 2-way referral (2 studies [30,31], 11%). The duration of the interventions ranged from 12 months (1 study [24], 6%), 6 months (10 studies [12,18,19,21,22,27-31], 56%), 3 months (4 studies [10,20,25,26], 22%) to 5 weeks (1 study [9], 6%), with 2 studies [6,23] (11%) not specifying the duration of the intervention.

### Control Group Interventions

The control group interventions in the included studies consisted of routine care, including routine risk assessment (1 study [12]), face-to-face education (12 studies [9,10,18-20,23-25,28-31]), and telephone follow-up (14 studies [6,18-22,24-31]). The intervention content primarily involved face-to-face self-management health education provided by nurses at discharge, as well as follow-up and self-management guidance via telephone by nurses after discharge.

### Evaluation Indicators and Tools

The evaluation indicators in the included studies encompassed four categories: (1) foot care behavior (5 studies [9,12,25,30,31], 28%), diabetes self-management ability (9 studies [6,10,19,20,22,24,27-29], 50%), general self-management ability (1 study [18], 6%), and specific behaviors (3 studies [21,23,26], 17%). The tools used to assess foot care behavior included the DFCB questionnaire [12], DFSBS [9], Self-management Model Questionnaire [25], and DFSQ-UMA [30,31]. The assessment content focused on whether patients regularly checked their feet, the condition of their foot care, and whether they selected appropriate footwear. The tools used to evaluate diabetes self-management ability included the QAD [10], Diabetes Self-management Behavior Scale [6,28], SDSCA [19,24,29], and 2-DSCS [20,22,27]. The assessment content primarily covered blood glucose monitoring, proper diet, regular exercise, foot care, and medication adherence. The tool used to assess self-management ability was the Self-care Agency Scale [18], which evaluated a patient’s general disease self-management ability, including 4 dimensions, that is, self-care skills, self-care responsibility, self-concept, and health knowledge level. Specific behaviors reflecting the patient’s self-management status, consisted of blood glucose monitoring, foot examination, and regular follow-up visits in the included studies [21,23,26].

### Effects of Digital Intelligent Interventions on Self-Management of Patients With DF

Among the 18 included studies, 17 studies [6,9,12,18-31] (94%) indicated that, compared with routine care, digital intelligent interventions significantly improved the self-management levels of patients with DF. However, 1 study [10] (6%) showed that the effects of digital intelligent interventions were not significantly different from those of routine care. Based on the results of the included studies, when using WeChat for intervention, a 6-month intervention for patients with Wagner grade 0 [27,28] or 1-2 [19], or a 3-month [20] or 6-month [22] intervention for patients without classification, can significantly improve patients’ self-management ability in diabetes. When using an electronic platform for intervention, a 6-month intervention for patients with Wagner grade 0 [20] or 0-2 [30,31] can significantly enhance patients’ diabetes self-management or foot care levels. When using an app for intervention, a 5-week [9] or 6-month [21] intervention for patients with Wagner grade 0 can improve patients’ foot care behaviors, self-monitoring of blood glucose, and regular return visits; a 3-month [26] intervention for patients with Wagner grade 0-3 can improve behaviors of blood glucose monitoring and regular dressing changes, but had no significant effect on regular follow-up visits; while a 3-month [10] intervention for patients with Wagner grade 0 cannot significantly improve their diabetes self-management ability. Using smartphone thermography [10] for a 6-month intervention for patients with Wagner grade 0 can significantly improve foot care behaviors. Using WeChat combined with app [18] for a 6-month intervention for patients with Wagner grades 3-4 can significantly improve general self-management ability. Using an electronic platform combined with app [24] for a 12-month intervention for patients with Wagner grade 0 can significantly improve their diabetes self-management ability. Using WeChat combined with electronic platform [25] for a 3-month intervention for unclassified patients can significantly improve foot care behaviors.

## Discussion

### Principal Findings

A total of 18 RCTs were included in this systematic review, all of which were assessed as high-risk studies. Methodological deficiencies in the included studies primarily included unclear details of randomization, lack of allocation concealment before grouping, absence of blinding, failure to conduct ITT analysis, high rates of missing data and loss to follow-up, absence of standardized assessment tools, unclear assessment timing, potential awareness of intervention status among assessment personnel, and failure to report total scores of scales. Due to the significant heterogeneity among studies, we conducted a qualitative synthesis of the included studies. The results showed that overall, digital intelligent interventions improved self-management ability among patients with DF. However, the target populations, intervention forms, intervention durations, and assessment tools vary across different studies, and there is currently no standardized intervention paradigm in this field.

The most noteworthy point is that, among the 18 studies, only 1 study [10] did not show a significant effect of digital intelligent intervention, and the reasons for this phenomenon need to be analyzed in depth. In that study [10], the participants were patients with Wagner grade 0, the intervention form was an app (used for foot care education), the intervention duration was 3 months, and QAD was used to assess the patients’ diabetes self-management ability. In another study [9], where the app intervention (used for foot care education, foot care reminders, and foot care behavior supervision) also targeted patients with Wagner grade 0, but the duration was 5 weeks, the DFSBS assessment showed significant improvements in patients’ foot care behaviors. This seems to illustrate the importance of the intervention components, suggesting that even with a shorter intervention duration, focusing on more aspects of self-management (not just education, but also reminders and supervision) can lead to better outcomes. In 1 study [21], a 6-month intervention with the app (used for foot care education, blood glucose, and foot monitoring) in patients with Wagner grade 0, improvements in self-monitoring of blood glucose and regular visits were observed. Similarly, for app interventions targeting patients with Wagner grade 0, the outcomes are better when the intervention included more components and lasted longer. When we broaden our perspective beyond just app interventions and patients with Wagner grade 0, and compare this study [10] with others, we find that regardless of the intervention form (WeChat, App, electronic platforms, or mixed interventions), the intervention duration (shorter or longer), and target population (Wagner grade 0 or Wagner grade ≥1), it seems that as long as the intervention is not limited to health education alone (but also includes reminders, supervision, or follow-ups, etc), the significant effects of digital intelligent interventions can be observed.

The deeper reasons for these results need to be discussed. Health education does have an effect on self-management behaviors of patients with diabetes, as existing studies [32,33] have confirmed. However, our results suggest that, compared with conventional face-to-face health education, health education delivered solely through digital intelligent means (such as apps) does not show more significant effects than traditional interventions. A possible reason is that, although apps can overcome the boundaries of time and space, allowing patients to receive health education anytime and anywhere, such interventions do not guarantee that patients will actively engage with the app for health education. In such cases, the intervention may be less effective than the conventional 1-time “forced” face-to-face health education during discharge. In other studies, the inclusion of intervention components, such as reminders, supervision, and follow-up, helped to enhance patients’ adherence to using digital intelligent tools for health education. At the same time, these interventions directly reminded and supervised patients in implementing self-management behaviors, enabling the real-time, dynamic, and personalized features of digital intelligent interventions to be fully used, resulting in significantly better outcomes compared with conventional interventions.

This provides us with an important insight that it is not only the functions of digital intelligent tools themselves that are important, but it seems even more crucial to focus on how to attract users to continuously use these tools in order to fully leverage the advantages of digital intelligent technology. In the included studies, the methods used to remind and encourage patients to use the tools included setting up learning contracts [9] and real-time reminders in WeChat groups [18,21,27,28]. While these measures achieved good results, there was a lack of (or no report on) efforts made to enhance the attractiveness of the digital intelligent tools themselves. Factors that influence people’s use of digital intelligent tools have been explored in previous studies. One study [34] explored the technology adoption intention of users of smart wearable health technologies and identified the key factors affecting user technology adoption. The research [34] found that the more the user’s experience of smart health technology aligns with personal needs, the more positive the user’s emotional response, which in turn promotes higher user engagement. In addition, the operability and interaction design of the technology (such as the intuitiveness and ease of use of the interface) are also important factors in determining user engagement [34]. Based on this, the following strategies can be adopted to improve user engagement with digital intelligent tools. First, the users’ experience should be personalized. Personalization can strengthen users’ emotional attachment to the technology because they feel that the tool is more closely aligned with their lives, which in turn increases engagement and ultimately promotes usage [35]. Specific measures include (1) offering customized features, that is, developing features that cater to specific health goals and needs; for example, allow users to customize exercise plans, track specific health indicators (eg, blood sugar levels), or set personalized reminders (eg, foot check-ups). (2) Developing adaptive interfaces [36], that is, providing adaptive interfaces that change based on user preferences, usage patterns, and health progress. This can also include adjusting the complexity of information based on the user’s knowledge level and health literacy. Second, the focus should be on intuitive and user-friendly design. A user-friendly design increases the likelihood of long-term engagement because users feel more confident and comfortable when using the device, encouraging continued usage [37]. Specific measures include (1) simple and clear interface (ie, ensure the interface is simple, navigation is easy, and information is clear and straightforward; make sure buttons, menus, and options are simple and intuitive, reducing the complexity of setup procedures); (2) user testing and feedback (ie, continuously improve the design through user feedback; conduct usability tests to identify potential barriers in the interface and simplify them). Third, emotional engagement and motivational incentives are also crucial. Emotional engagement, driven by motivation and rewards, can strengthen the bond between users and the device, leading to higher usage frequency and adherence [38]. Specific measures include (1) gamification and reward mechanisms [39], that is, introduce gamification elements such as achievement badges, points, or milestones to recognize users’ health progress. These rewards can be linked to self-management behaviors (eg, monitoring blood sugar and performing foot care) or improvements in health indicators (eg, blood sugar levels and foot condition). (2) Social sharing features [40], that is, allow users to share their progress with friends or on social media. Social support can significantly increase motivation and promote engagement. Community-driven challenges (eg, foot care goals) can also enhance a sense of belonging and competition. Finally, continuous support and updates for the tool are essential. Ensure that the device is regularly updated, adding new features and improvements based on user feedback. Software updates should not only address issues but also enhance overall functionality, improving the value of the user experience [40]. Ongoing support and updates increase the reliability of the technology and the commitment to the users, thus encouraging long-term adoption.

The final aspect worth discussing is the regional distribution of the studies. The 18 studies we included were all conducted in limited-income countries (Indonesia, Brazil, Malaysia, and China), with 15 of them being conducted in China. One of the reasons for the high number of studies from China could be the inclusion of 4 Chinese language databases in our search. However, this is not the only or primary reason, as we also conducted systematic searches across 7 authoritative English language databases. In addition, after carefully reviewing the search terms, we consider our search to be reasonably comprehensive. Therefore, it is more likely that the regional distribution of the studies reflects the current global distribution of the application of digital intelligent technologies in self-management of patients with DF. Although digital intelligent technologies have been rapidly developed in high-income countries, this study focuses specifically on their application in the self-management of patients with DF. Compared with high-income countries, limited-income countries, due to relatively lower economic levels and uneven distribution of medical resources, face significant challenges in diabetes management and early prevention of DF, resulting in a higher number of patients with DF and consequently, a greater focus on self-management research within this patient group [41]. Similarly, the possible reason for the large number of studies from China is that it has the world’s largest population and the highest number of patients with diabetes [42]. In addition, due to the vast territory of China and the uneven distribution of health care resources [43], managing diabetes and preventing DF pose significant challenges, resulting in a higher number of patients with DF [44] and, consequently, more research on their self-management.

### Limitations

First, the overall quality of the included studies is relatively low, with several methodological flaws that may affect the reliability of the results. Second, due to significant heterogeneity among the included studies, a meta-analysis could not be conducted. Although most included studies indicated that digital intelligent interventions were effective, the lack of quantitatively synthesized results hinders the determination of the optimal intervention strategy, such as the best form of intervention or the ideal duration of the intervention. Finally, although we made efforts to ensure the comprehensiveness of the search terms (keywords and their synonyms) and databases (4 Chinese databases and 7 English databases), the majority of the included studies were conducted in China, which may introduce cultural and regional biases and limit the generalizability of the findings.

### Conclusions

In this systematic review, evidence suggested that digital intelligent interventions can improve self-management behaviors and capabilities in patients with DF. However, due to the overall low quality of the included studies, current evidence should be interpreted and applied with caution. At present, this field is still in the exploratory stage, with significant heterogeneity among different studies and a lack of consensus on intervention strategies, necessitating further exploration tailored to different populations. Future RCTs with large sample sizes and rigorous design are needed to develop high-quality evidence and determine the optimal intervention strategies. In addition, in order to fully leverage the advantages of digital intelligent technology, the human-centered design of digital intelligent tools, including personalization, ease of use, and intuitiveness, should also be given attention, alongside their health management functions.

## Appendix

Multimedia Appendix 1 Search strategy of each database and details of the included studies.

Multimedia Appendix 2 PRISMA checklist.

Multimedia Appendix 3 Digital intelligent intervention forms, components, and durations of the included studies.

## Abbreviations

DF: diabetic foot
DFCB: diabetic foot care behavior
DFSBS: Diabetic Foot Selfcare Behavior Scale
DFSQ-UMA: Diabetic Foot Self-Care Questionnaire of the University of Malaga
DM: diabetes mellitus
ITT: intention-to-treat
IWGDF: International Working Group on the Diabetic Foot
PICOS: P: population, I: intervention, C: comparison, O: outcome, S: study design
PRISMA: Preferred Reporting Items for Systematic Reviews and Meta-Analyses
QAD: Diabetes Self-care Activities Questionnaire
RCT: randomized controlled trial
SDSCA: Summary of Diabetes Self-Care Activities
2-DSCS: Type 2 Diabetes Self-Care Scale

## Data Availability

Datasets generated and analyzed during the study are available from the corresponding author upon reasonable request.

## References

[1] van Netten JJ, Bus SA, Apelqvist J, Chen P, Chuter V, Fitridge R, Game F, Hinchliffe RJ, Lazzarini PA, Mills J, Monteiro-Soares M, Peters EJG, Raspovic KM, Senneville E, Wukich DK, Schaper NC, International Working Group on the Diabetic Foot (2024). Definitions and criteria for diabetes-related foot disease (IWGDF 2023 update). Diabetes Metab Res Rev.

[2] Bakker K, Apelqvist J, Lipsky BA, Van Netten JJ, International Working Group on the Diabetic Foot (2016). The 2015 IWGDF guidance documents on prevention and management of foot problems in diabetes: development of an evidence-based global consensus. Diabetes Metab Res Rev.

[3] Schaper NC, van Netten JJ, Apelqvist J, Bus SA, Fitridge R, Game F, Monteiro-Soares M, Senneville E, IWGDF Editorial Board (2024). Practical guidelines on the prevention and management of diabetes-related foot disease (IWGDF 2023 update). Diabetes Metab Res Rev.

[4] Schaper NC, Van Netten JJ, Apelqvist J, Lipsky BA, Bakker K, International Working Group on the Diabetic Foot (IWGDF) (2017). Prevention and management of foot problems in diabetes: a summary guidance for daily practice 2015, based on the IWGDF guidance documents. Diabetes Res Clin Pract.

[5] Costa IG, Tregunno D, Camargo-Plazas P (2020). I cannot afford off-loading boots: perceptions of socioeconomic factors influencing engagement in self-management of diabetic foot ulcer. ANS Adv Nurs Sci.

[6] Zhang F (2022). Analysis of the effects of continuous nursing based on WeChat platform on self-management behavior and self-efficacy of patients with diabetic foot. Harbin Medical Journal.

[7] Zhang W, Zhang C, Cao L, Liang F, Xie W, Tao L, Chen C, Yang M, Zhong L (2023). Application of digital-intelligence technology in the processing of Chinese materia medica. Front Pharmacol.

[8] Wang Y, Xiong X, Liu M, Yang Y, Liu S, Chen H, Zhang M, Xiang Q (2024). Application progress of digital and intelligent intervention technology in the mental disorders of patients with coronary heart disease. Chinese Journal of Nursing, Review.

[9] Firdaus MKZH, Jittanoon P, Boonyasopun U, Che Hasan MK (2023). The effect of mHealth program on behavior modification and health outcomes among patients with diabetes: a randomized controlled trial study. Belitung Nurs J.

[10] Marques ADB, Moreira TMM, Mourão Luana Feitosa, Florêncio Raquel Sampaio, Cestari VRF, Garces TS, Bruno NA (2023). Mobile application for adhering to diabetic foot self-care: randomized controlled clinical trial. Comput Inform Nurs.

[11] Moulaei K, Malek M, Sheikhtaheri A (2021). A smart wearable device for monitoring and self-management of diabetic foot: a proof of concept study. Int J Med Inform.

[12] Qin Q, Oe M, Nakagami G, Kashiwabara K, Sugama J, Sanada H, Jais S (2023). The effectiveness of a thermography-driven preventive foot care protocol on the recurrence of diabetic foot ulcers in low-medical resource settings: An open-labeled randomized controlled trial. Int J Nurs Stud.

[13] Higgins JPT, Chandler TJ, Cumpston J, Li T, Page MJ, Welch VA (2023). Cochrane Handbook for Systematic Reviews of Interventions version 6.4.

[14] Toobert DJ, Hampson SE, Glasgow RE (2000). The summary of diabetes self-care activities measure: results from 7 studies and a revised scale. Diabetes Care.

[15] Lee NP, Fisher WP (2002). Evaluation of the diabetes self care scale: an illustration of the Rasch model of measurement. J Nurs Meas.

[16] Navarro-Flores E, Morales-Asencio JM, Cervera-Marín José Antonio, Labajos-Manzanares MT, Gijon-Nogueron G (2015). Development, validation and psychometric analysis of the diabetic foot self-care questionnaire of the University of Malaga, Spain (DFSQ-UMA). J Tissue Viability.

[17] Sterne JAC, Savović J, Page MJ, Elbers RG, Blencowe NS, Boutron I, Cates CJ, Cheng H, Corbett MS, Eldridge SM, Emberson JR, Hernán Miguel A, Hopewell S, Hróbjartsson Asbjørn, Junqueira DR, Jüni Peter, Kirkham JJ, Lasserson T, Li T, McAleenan A, Reeves BC, Shepperd S, Shrier I, Stewart LA, Tilling K, White IR, Whiting PF, Higgins JPT (2019). RoB 2: a revised tool for assessing risk of bias in randomised trials. BMJ.

[18] Xu G, Liu M, Ju Y, Kong Y, Miao Y (2023). Application of Internet + follow-up management in patients with diabetic foot after tibial transverse bone transport. Tianjin Journal of Nursing.

[19] Zhang A, Xueqin H, Chunhong Y, Qiong W (2021). The application effect of goal-oriented hospital-community linkage continuous nursing in patients with diabetic foot. Proceeding of Clinical Medicine.

[20] Chu, L, Wang J, You Q, Fang Z, Dou L (2021). Continuous nursing pathway for diabetes foot based on WeChat platform. Journal of Clinical Medicine in Practice.

[21] Li K, Feng Y, Sun M, Cui Y, Gong X, Duan W, Xiao Y, Jiang L (2021). The self-influence of telemedicine system in the management of diabetic high risk foot. Electronic Journal of Foot and Ankle Surgery.

[22] Ye Q, Yu Y (2021). Study on the application effect of continuous care on WeChat platform in diabetic foot patients' self-management behavior. China & Foreign Medical Treatment.

[23] Qu Y (2020). Effects of transitional care based on WeChat APP on self-management and self-efficacy of patients with diabetic foot. The Medical Forum.

[24] Xie X, Wang X, Zhao D, Dong H, Zheng Z, Liu S, Li A, Ke J (2020). Application of internet plus blood glucose management mode in the education and management of diabetic foot patients. China Medical Herald.

[25] Xu, J (2019). Effect of extended care group combined with internet + on hospital discharge nursing for diabetic foot. Journal of Clinical Medicine in Practice.

[26] Liu Y (2019). Study on the advantages of managing patients with diabetic foot based on telemedicine system. University of Jinan.

[27] Liu J, Liu, X, Meng G, Tang J (2018). Study on self management behavior of diabetes foot in rural middle aged and elderly based on WeChat's continuous nursing. Medical Education Research and Practice.

[28] Tan X, Liu J, Wu J, Hong H, Liu C (2020). Effects of continuous nursing based on WeChat platform on self-management behavior and self-efficacy of patients with diabetic foot. International Journal of Nursing.

[29] Hu S (2018). Study on the effect of intelligent hierarchical management platform on self-management ability of patients with high-risk factors of diabetic foot. Ji'nan University.

[30] Bai J, Di R, Chen J (2022). Application of medical alliance co-management hierarchical diagnosis and treatment model in hospital-community comprehensive management of diabetic foot patients. Chinese Journal of Modern Nursing.

[31] Wang Y, Zhang M, Lyu F, Zheng X, Hu H, Lin T (2023). Observation on the effect of medical alliance co-management hierarchical diagnosis and treatment model applied to diabetic foot patients. Medical Innovation of China.

[32] Diriba DC, Leung DYP, Suen LKP (2024). Nurse-led self-management education and support programme on self-management behaviour and quality of life among adults with type 2 diabetes: A pilot randomized controlled trial. Int J Nurs Pract.

[33] Othman N, Al-Otaibi T, Halim MA, Said T, Elserwy N, Mahmoud F, Abduo H, Jahromi M, Nampoory N, Gheith OA (2024). Effect of repeated structured diabetes education on lifestyle knowledge and self-care diabetes management in kidney transplant patients with posttransplant diabetes. Exp Clin Transplant.

[34] Liu X (2024). The influence mechanism of smart wearable health technologies user technology adoption intention: based on the mediating role of user engagement.

[35] Rana A, Sanner S, Bouadjenek MR, Di Carlantonio R, Farmaner G (2024). User experience and the role of personalization in critiquing-based conversational recommendation. ACM Trans. Web.

[36] Wang W, Khalajzadeh H, Grundy J, Madugalla A, McIntosh J, Obie HO (2023). Adaptive user interfaces in systems targeting chronic disease: a systematic literature review. User Model User-Adap Inter.

[37] Najafi A, Letchumanan V (2011). Design and implementation of surepkay; a user friendly multimedia content management system for digital signage.

[38] Esmaeilzadeh P (2021). The influence of gamification and information technology identity on postadoption behaviors of health and fitness app users: empirical study in the United States. JMIR Serious Games.

[39] Bitrián P, Buil I, Catalán S (2021). Enhancing user engagement: The role of gamification in mobile apps. Journal of Business Research.

[40] Jia C, Qi H (2024). Users’ health information sharing behavior in social media: an integrated model. Humanit Soc Sci Commun.

[41] Swaminathan N, Awuah WA, Bharadwaj HR, Roy S, Ferreira T, Adebusoye FT, Ismail IFNB, Azeem S, Abdul-Rahman T, Papadakis M (2024). Early intervention and care for diabetic foot ulcers in low and middle income countries: addressing challenges and exploring future strategies: a narrative review. Health Sci Rep.

[42] Xu Y, Lu J, Li M, Wang T, Wang K, Cao Q, Ding Y, Xiang Y, Wang S, Yang Q, Zhao X, Zhang X, Xu M, Wang W, Bi Y, Ning G (2024). Diabetes in China part 1: epidemiology and risk factors. The Lancet Public Health.

[43] Zhang T, Xu Y, Ren J, Sun L, Liu C (2017). Inequality in the distribution of health resources and health services in China: hospitals versus primary care institutions. Int J Equity Health.

[44] Jiang YF, Wang X, Xia L, Fu X, Xu Z, Ran X, Yan L, Li Q, Mo Z, Yan Z, Ji Q, Li Q (2015). A cohort study of diabetic patients and diabetic foot ulceration patients in China. Wound Repair Regen.

