# Supplementary material for: Effects of Digital Intelligent Interventions on Self-Management of Patients With Diabetic Foot: Systematic Review
Source: J Med Internet Res. 2025 Mar 25;27:e64400. doi: 10.2196/64400 (PMC11979535; doi:10.2196/64400)
Supplement: Multimedia Appendix 1 [file jmir_v27i1e64400_app1.docx]

**Effects of digital intelligent interventions on self-management of patients with diabetic foot:**

**a systematic review**

**Table S1. Search strategy of each database**

| No. | Database | Search strategy | Number of articles |
| --- | --- | --- | --- |
| 1 | PubMed | ("Diabetic Foot"[Mesh] OR Diabetic Foot[Title/Abstract] OR Diabetic Feet[Title/Abstract] OR Diabetes foot[Title/Abstract] OR Diabetes feet[Title/Abstract] OR DF[Title/Abstract] OR Diabetes Mellitus Foot[Title/Abstract] OR (Diabetes-Related[Title/Abstract] AND Foot[Title/Abstract]) OR Diabetic Foot Ulcer[Title/Abstract] OR Foot Ulcer*[Title/Abstract] OR DFU[Title/Abstract] OR Foot Gangrene[Title/Abstract] OR Charcot Foot[Title/Abstract]) AND ((("Digital Health"[Mesh] OR "Digital Technology"[Mesh]) OR "Artificial Intelligence"[Mesh]) OR (Digital health[Title/Abstract] OR Intelligen*[Title/Abstract] OR Mobile health[Title/Abstract] OR mHealth[Title/Abstract] OR 'm-health'[Title/Abstract] OR eHealth[Title/Abstract] OR 'e-health'[Title/Abstract] OR telehealth[Title/Abstract] OR information[Title/Abstract] AND communication technology[Title/Abstract] OR information technology[Title/Abstract] OR health app[Title/Abstract] OR app[Title/Abstract] OR mobile application[Title/Abstract] OR phone[Title/Abstract] OR smartphone[Title/Abstract] OR mobile phone[Title/Abstract] OR cell phone[Title/Abstract] OR iphone[Title/Abstract] OR ipad[Title/Abstract] OR ipod[Title/Abstract] OR wearable[Title/Abstract] OR sensor[Title/Abstract] OR portable[Title/Abstract] OR tablet[Title/Abstract] OR Android[Title/Abstract] OR iOS[Title/Abstract] OR web-based[Title/Abstract] OR web[Title/Abstract] OR website[Title/Abstract] OR online[Title/Abstract] OR internet[Title/Abstract] OR Social media[Title/Abstract] OR Facebook[Title/Abstract] OR Twitter[Title/Abstract] OR web-sites[Title/Abstract] OR TV[Title/Abstract] OR SMS[Title/Abstract] OR Message[Title/Abstract] OR Email[Title/Abstract] OR video[Title/Abstract] OR phone platform[Title/Abstract] OR network platform[Title/Abstract] OR digital media[Title/Abstract] OR multimedia[Title/Abstract])) AND (("Self-Management"[Mesh]) OR (Self assessment[Title/Abstract] OR self management[Title/Abstract] OR self manage*[Title/Abstract] OR self monitor*[Title/Abstract] OR Self care[Title/Abstract])) | 101 |
| 2 | Web of Science | Diabetic Foot OR Diabetic Feet OR Diabetes foot OR Diabetes feet OR DF OR Diabetes Mellitus Foot OR (Diabetes-Related AND Foot) OR Diabetic Foot Ulcer OR Foot Ulcer* OR DFU OR Foot Gangrene OR Charcot Foot (Title) and Digital health OR Intelligen* OR Mobile health OR mealth OR 'm-health' OR eearth OR 'e-health' OR telehealth OR information and communication technology OR information technology OR health app OR app OR mobile application OR phone OR smartphone OR mobile phone OR cell phone OR iphone OR ipad OR ipod OR wearable OR sensor OR portable OR tablet OR Android OR iOS OR web-based OR web OR website OR online OR internet OR Social media OR Facebook OR Twitter OR web-sites OR TV OR SMS OR Message OR Email OR video OR phone platform OR network platform OR digital media OR multimedia (Topic) and Self assessment OR self management OR self manage* OR self monitor* OR Self care (Title) | 53 |
| 3 | Embase | ('diabetic foot':ti,ab,kw OR 'diabetic feet':ti,ab,kw OR 'diabetes foot':ti,ab,kw OR 'diabetes feet':ti,ab,kw OR df:ti,ab,kw OR 'diabetes mellitus foot':ti,ab,kw OR ('diabetes related':ti,ab,kw AND foot:ti,ab,kw) OR 'diabetic foot ulcer':ti,ab,kw OR 'foot ulcer*':ti,ab,kw OR dfu:ti,ab,kw OR 'foot gangrene':ti,ab,kw OR 'charcot foot':ti,ab,kw) AND (('digital health':ti,ab,kw OR intelligen*:ti,ab,kw OR 'mobile health':ti,ab,kw OR mealth:ti,ab,kw OR 'm-health':ti,ab,kw OR eearth:ti,ab,kw OR 'e-health':ti,ab,kw OR telehealth:ti,ab,kw OR information:ti,ab,kw) AND 'communication technology':ti,ab,kw OR 'information technology':ti,ab,kw OR 'health app':ti,ab,kw OR app:ti,ab,kw OR 'mobile application':ti,ab,kw OR phone:ti,ab,kw OR smartphone:ti,ab,kw OR 'mobile phone':ti,ab,kw OR 'cell phone':ti,ab,kw OR iphone:ti,ab,kw OR ipad:ti,ab,kw OR ipod:ti,ab,kw OR wearable:ti,ab,kw OR sensor:ti,ab,kw OR portable:ti,ab,kw OR tablet:ti,ab,kw OR android:ti,ab,kw OR ios:ti,ab,kw OR 'web based':ti,ab,kw OR web:ti,ab,kw OR website:ti,ab,kw OR online:ti,ab,kw OR internet:ti,ab,kw OR 'social media':ti,ab,kw OR facebook:ti,ab,kw OR twitter:ti,ab,kw OR 'web sites':ti,ab,kw OR tv:ti,ab,kw OR sms:ti,ab,kw OR message:ti,ab,kw OR email:ti,ab,kw OR video:ti,ab,kw OR 'phone platform':ti,ab,kw OR 'network platform':ti,ab,kw OR 'digital media':ti,ab,kw OR multimedia:ti,ab,kw) AND ('self assessment':ti,ab,kw OR 'self management':ti,ab,kw OR 'self manage*':ti,ab,kw OR 'self monitor*':ti,ab,kw OR 'self care':ti,ab,kw) | 124 |
| 4 | Cumulative Index to Nursing and Allied Health Literature | SU ( Diabetic Foot OR Diabetic Feet OR Diabetes foot OR Diabetes feet OR DF OR Diabetes Mellitus Foot OR (Diabetes-Related AND Foot) OR Diabetic Foot Ulcer OR Foot Ulcer* OR DFU OR Foot Gangrene OR Charcot Foot ) AND SU ( Digital health OR Intelligen* OR Mobile health OR mHealth OR 'm-health' OR eHealth OR 'e-health' OR telehealth OR information and communication technology OR information technology OR health app OR app OR mobile application OR phone OR smartphone OR mobile phone OR cell phone OR iphone OR ipad OR ipod OR wearable OR sensor OR portable OR tablet OR Android OR iOS OR web-based OR web OR website OR online OR internet OR Social media OR Facebook OR Twitter OR web-sites OR TV OR SMS OR Message OR Email OR video OR phone platform OR network platform OR digital media OR multimedia ) AND SU ( Self assessment OR self management OR self manage* OR self monitor* OR Self care ) Retrieval mode - Intelligent text search | 53 |
| 5 | PsycINFO | SU (Diabetic Foot OR Diabetic Feet OR Diabetes foot OR Diabetes feet OR DF OR Diabetes Mellitus Foot OR (Diabetes-Related AND Foot) OR Diabetic Foot Ulcer OR Foot Ulcer* OR DFU OR Foot Gangrene OR Charcot Foot ) AND SU ( Digital health OR Intelligen* OR Mobile health OR mHealth OR 'm-health' OR eHealth OR 'e-health' OR telehealth OR information and communication technology OR information technology OR health app OR app OR mobile application OR phone OR smartphone OR mobile phone OR cell phone OR iphone OR ipad OR ipod OR wearable OR sensor OR portable OR tablet OR Android OR iOS OR web-based OR web OR website OR online OR internet OR Social media OR Facebook OR Twitter OR web-sites OR TV OR SMS OR Message OR Email OR video OR phone platform OR network platform OR digital media OR multimedia ) AND SU ( Self assessment OR self management OR self manage* OR self monitor* OR Self care ) Retrieval mode - Intelligent text search | 11 |
| 6 | Cochrane Central Register of Controlled Trials | Diabetic Foot OR Diabetic Feet OR Diabetes foot OR Diabetes feet OR DF OR Diabetes Mellitus Foot OR (Diabetes-Related AND Foot) OR Diabetic Foot Ulcer OR Foot Ulcer* OR DFU OR Foot Gangrene OR Charcot Foot in Title Abstract Keyword AND Digital health OR Intelligen* OR Mobile health OR mHealth OR 'm-health' OR eHealth OR 'e-health' OR telehealth OR information and communication technology OR information technology OR health app OR app OR mobile application OR phone OR smartphone OR mobile phone OR cell phone OR iphone OR ipad OR ipod OR wearable OR sensor OR portable OR tablet OR Android OR iOS OR web-based OR web OR website OR online OR internet OR Social media OR Facebook OR Twitter OR web-sites OR TV OR SMS OR Message OR Email OR video OR phone platform OR network platform OR digital media OR multimedia in Title Abstract Keyword AND Self assessment OR self management OR self manage* OR self monitor* OR Self care in Title Abstract Keyword | 290 |
| 7 | ProQuest | subject(Diabetic Foot OR Diabetic Feet OR Diabetes foot OR Diabetes feet OR DF OR Diabetes Mellitus Foot OR (Diabetes-Related AND Foot) OR Diabetic Foot Ulcer OR Foot Ulcer* OR DFU OR Foot Gangrene OR Charcot Foot) AND subject(Digital health OR Intelligen* OR Mobile health OR mHealth OR 'm-health' OR eHealth OR 'e-health' OR telehealth OR information AND communication technology OR information technology OR health app OR app OR mobile application OR phone OR smartphone OR mobile phone OR cell phone OR iphone OR ipad OR ipod OR wearable OR sensor OR portable OR tablet OR Android OR iOS OR web-based OR web OR website OR online OR internet OR Social media OR Facebook OR Twitter OR web-sites OR TV OR SMS OR Message OR Email OR video OR phone platform OR network platform OR digital media OR multimedia) AND summary(Self assessment OR self management OR self manage* OR self monitor* OR Self care) | 46 |
| 8 | China National Knowledge Internet | （主题：糖尿病足 + 足溃疡 + 足疾病 + 足病 + 足部溃疡 + 糖尿病坏疽 + 糖尿病血管病变 + 糖尿病神经病变+ 糖尿病性足溃疡 + 糖尿病肢端坏疽 + 糖尿病溃疡 + 足创面 +夏科氏足）AND（主题：数字+ 智能 + 移动+ 电子健康 + 远程医疗 +信息通讯技术 + 信息与通讯技术 + 信息和通讯技术 + 信息技术 + 互联网 + 网络 + 在线 + 线上 + 网站 + 平台 + 应用程序 + 应用软件 + APP + 电话 + 手机）AND（主题：自我管理 + 自我护理 + 自我照护 + 自我保健 + 自我照顾） | 213 |
| 9 | WanFang | 题名或关键词:(糖尿病足 OR 足溃疡 OR 足疾病 OR 足病 OR 足部溃疡 OR 糖尿病坏疽 OR 糖尿病血管病变 OR 糖尿病神经病变OR 糖尿病性足溃疡 OR 糖尿病肢端坏疽 OR 糖尿病溃疡 OR 足创面) and 题名或关键词:(数字 OR 智能 OR 移动 OR 电子 OR 远程 OR 互联网 OR 网络 OR 在线 OR 线上 OR 网站 OR 平台 OR 软件 OR APP OR 电话 OR 手机 OR 短信 OR 视频) and 题名或关键词:(自我管理 OR 自我护理 OR 自我照护 OR 自我保健 OR 自我照顾) | 28 |
| 10 | China Science and Technology Journal Database | ((((((((((((题名或关键词=糖尿病足 OR 题名或关键词=足溃疡) OR 题名或关键词=足疾病) OR 题名或关键词=足病) OR 题名或关键词=足部溃疡) OR 题名或关键词=糖尿病坏疽) OR 题名或关键词=糖尿病血管病变) OR 题名或关键词=糖尿病神经病变OR 糖尿病性足溃疡) OR 题名或关键词=糖尿病肢端坏疽) OR 题名或关键词=糖尿病溃疡) OR 题名或关键词=足创面 OR夏科氏足) AND ((((((((((((((((题名或关键词=数字 OR 题名或关键词=智能) OR 题名或关键词=移动) OR 题名或关键词=电子) OR 题名或关键词=远程) OR 题名或关键词=互联网) OR 题名或关键词=网络) OR 题名或关键词=在线) OR 题名或关键词=线上) OR 题名或关键词=网站) OR 题名或关键词=平台) OR 题名或关键词=软件) OR 题名或关键词=APP) OR 题名或关键词=电话) OR 题名或关键词=手机) OR 题名或关键词=短信) OR 题名或关键词=视频)) AND ((((题名或关键词=自我管理 OR 题名或关键词=自我护理) OR 题名或关键词=自我照护) OR 题名或关键词=自我保健) OR 题名或关键词=自我照顾)) | 21 |
| 11 | SinoMed | ( "糖尿病足"[摘要:智能] OR "足溃疡"[摘要:智能] OR "足疾病"[摘要:智能] OR "足病"[摘要:智能] OR "足部溃疡"[摘要:智能] OR "糖尿病坏疽"[摘要:智能] OR "糖尿病血管病变"[摘要:智能] OR "糖尿病神经病变OR 糖尿病性足溃疡"[摘要:智能] OR "糖尿病肢端坏疽"[摘要:智能] OR "糖尿病溃疡"[摘要:智能] OR "足创面 OR夏科氏足"[摘要:智能]) AND( "数字"[摘要:智能] OR "智能"[摘要:智能] OR "移动"[摘要:智能] OR "电子"[摘要:智能] OR "远程"[摘要:智能] OR "互联网"[摘要:智能] OR "网络"[摘要:智能] OR "在线"[摘要:智能] OR "线上"[摘要:智能] OR "网站"[摘要:智能] OR "平台"[摘要:智能] OR "软件"[摘要:智能] OR "APP"[摘要:智能] OR "电话"[摘要:智能] OR "手机"[摘要:智能] OR "短信"[摘要:智能] OR "视频"[摘要:智能]) AND( "自我管理"[摘要:智能] OR "自我护理"[摘要:智能] OR "自我照护"[摘要:智能] OR "自我保健"[摘要:智能] OR "自我照顾"[摘要:智能]) | 139 |

***Notes: This Supplementary file provides the search strategy details, performed February 6th, 2025.***

**Table S2. Details of the included studies.**

| Author(s), year, | Country | Included centers | Participant | | | | | | Digital intelligent intervention | | Control intervention | | Use of ITT | Outcome measurements | Results of interest (I VS. C) |
| --- | --- | --- | --- | --- | --- | --- | --- | --- | --- | --- | --- | --- | --- | --- | --- |
|  |  |  | Type^a^ | No. (I/C)^b^ | Age Mean (SD) | | Male [n (%)] | | Content | Duration | Content | Duration |  |  |  |
|  |  |  |  |  | I | C | I | C |  |  |  |  |  |  |  |
| Qin 2023[12] | Indonesia | Two | Diabetic patients with a history of DFU (Wagner grade 0) | 60/60 | 61.7 (10.78) | 59.7 (10.61) | 29 (48.3) | 29 (48.3) | Smartphone thermography evaluation + Personalized foot care and education | 6 m | Usual care and education using a booklet | 6 m | Y | Foot care behavior (modified Indonesian version of the DFCB questionnaire) | LSM ± SE: 67.5±1.20 VS. 51.9 ±1.52 (*p* < 0.001) |
| Marques 2023[10] | Brazil | One | T2DM medical diagnosis and foot at risk (Wagner grade 0) | 20/22 | - | - | - | - | Standard nursing consultations + application use | 3 m | Standard nursing consultations | 3 m | N | Self-care activities with diabetes (QAD) | Mean ± SD: 4.52±0.78 VS. 4.51 ±0.93 (*p*=0.987) |
| Firdaus 2023[9] | Malaysia | One | Last two readings of A1c more than 6.5, has at least Stage 1 or above risk of foot ulcer development (Wagner grade 0) | 29/29 | 50.03 (8.03) | 57.59 (7.18) | 9 (31.0) | 11 (37.9) | Diabetic Care Self Management Mobile Health Application Program | 5 w | Standard care | 5 w | N | Foot care behavior (DFSBS) | After controlling for age, monthly income, and family history, the foot care behavior of the intervention group was significantly better than that of the control group (F=30.374, p <0.01). |
| Xu 2023[18] | China | One | Patients with DF discharged after unilateral transverse tibial bone transport, Wagner grade was 3-4 | 21/21 | 64.5 (8.6) | 65.8 (8.4) | 18 (85.7) | 19 (90.5) | "Internet +" follow-up nursing intervention (WeChat group, WeChat Official Account, and Blood Glucose Monitoring App) | 6 m | Health guidance was given before discharge and routine telephone follow-up | 6 m | Y | General self-management ability (Chinese version of Self-care Agency Scale) | The scores of self-care skills, self-care responsibility, self-concept, and health knowledge in the intervention group were higher than those in the control group (*p*<0.05). |
| Zhang 2022[6] | China | One | Patients with DF with Wagner grade 0 | 46/46 | 65.69(3.78) | 65.69(3.78) | 25 (54.3) | 26 (56.5) | Transitional care based on WeChat platform (WeChat patient group, WeChat Official Account, educational knowledge in the form of text, pictures, cartoons, audio, and video) | - | Telephone follow-up was conducted after discharge | - | Y | Diabetes self-management ability (Diabetes Self-management Behavior Scale) | Mean ± SD: 65.11±6.15 VS. 54.26±7.94 (*p*＜0.001) |
| Zhang 2021[19] | China | One | Patients with DF with Wagner grade 1-2 | 43/43 | 64.42(4.20) | 66.31(4.56) | 27 (62.8) | 24 (55.8) | Goal-oriented hospital-community linkage continuous nursing care (WeChat Official Account, WeChat patient group) | 6 m | Health guidance was given before discharge and regular telephone follow-up after discharge | 6 m | Y | Diabetes self-management ability (SDSCA) | Mean ± SD: 58.65±5.46 VS. 53.44 ±5.81 (*p*＜0.001) |
| Chu 2021[20] | China | One | Patients with DF | 60/60 | 51.49(12.73) | 52.42(11.40) | 37 (61.67) | 33 (55.00) | WeChat platform follow-up and continuous care (WeChat Official Account, WeChat patient group) | 12 w | Routine telephone follow-up and outpatient education | 12 w | Y | Diabetes self-management ability (2-DSCS) | The scores of self-management ability in all dimensions (prevention and treatment of high and low blood glucose, foot care, blood glucose monitoring, reasonable diet, medical compliance, and regular exercise) in the intervention group were higher than those in the control group (*p* < 0.05). |
| Li 2021[21] | China | One | Patients at high risk of DF (Wagner grade 0) | 58/58 | - | - | 31 (53.4) | 35 (60.3) | The diabetic foot App was used for remote management after discharge | 6 m | Telephone follow-up | 6 m | Y | Self-monitoring of blood glucose and regular return visit | N (%) Self-monitoring of blood glucose: 36 (62.1) VS. 18(31.0) (p < 0.05); Regular return visit: n (%) 49 (84.5) VS. 33 (56.9) (p < 0.05) |
| Ye 2021[22] | China | One | Patients with DF | 29/29 | 62.9 (9.4) | 63.2 (8.4) | 18 (62.1) | 15 (51.7) | Transitional care based on WeChat platform (WeChat Official Account, WeChat patient group) | 6 m | Routine nursing follow-up | 6 m | Y | Diabetes self-management ability (2-DSCS) | The scores of medication, exercise, diet, blood glucose measurement, and foot care in the intervention group were significantly higher than those in the control group (*p*< 0.05). |
| Qu 2020[23] | China | One | Patients with type 2 diabetic foot with Wagner grade 1-4 | 40/40 | 64.5 (5.3) | 64.0 (4.5) | 28 (70) | 30 (75) | Routine nursing + Continuing care by WeChat | - | Routine nursing | - | Y | Listen to a lecture on diabetes, discuss treatment options with doctor, self-monitoring of blood glucose, remeasuring blood glucose at the hospital, master the method of foot examination, master the method of insulin injection | The proportion of self-management ability indicators in the intervention group was higher than that in the control group (*p*<0.05). |
| Xie 2020[24] | China | One | Patients with DF with Wagner grade 0 | 100/95 | 61.1 (7.3) | 61.9 (6.8) | 48 (48) | 48 (50.5) | Internet+ blood glucose management (Blood glucose management platform + Blood Glucose Manager App) | 12 m | Conventional blood glucose management | 12 m | Y | Diabetes self-management ability (SDSCA) | The scores of all dimensions (healthy diet, exercise, self-monitoring of blood glucose, monitoring of blood glucose according to medical advice, self-examination of feet, and medication) in the intervention group were significantly higher than those in the control group (*p*< 0.01). |
| Xu 2019[25] | China | One | Patients with DF | 46/46 | 54.86(6.07) | 53.15 (5.16) | 24 (52.2) | 25 (54.3) | Extended care group combined with "Internet+" mode (WeChat, SMS, QQ and other Internet communication tools, Internet extended nursing platform) | 3 m | Routine nursing | 3 m | Y | Foot care behaviors (Self-management Model Questionnaire) | Mean ± SD: 87.36±9.27 VS. 71.68±9.68 (*p*<0.05) |
| Liu 2019[26] | China | One | Patients with DF (Wagner grade 0-3) | 26/32 | 64.73(11.85) | 65.09(11.54) | 14 (43.75) | 18 (69.23) | Telephone follow-up after discharge + DF App management | 3 m | Telephone follow-up after discharge | 3 m | Y | Blood glucose monitoring, regular follow-up visits, regular dressing changes | N (%): Blood glucose monitoring: 15 (57.69) VS. 10 (31.25) (*p*=0.043); Regular follow-up visits: 16 (61.54) VS. 12 (37.50) (*p*=0.068); Regular dressing changes: 22 (84.62) VS. 18 (56.25) (*p*=0.020) |
| Liu 2018[27] | China | One | Patients with DF with Wagner grade 0 | 90/90 | 59.8 (9.6) | 60.2 (9.3) | 62 (62) | 64 (64) | Transitional care based on WeChat platform (WeChat group, educational knowledge in the form of text, pictures, cartoons, and video) | 6 m | Telephone follow-up | 6 m | N | Diabetes self-management ability (2-DSCS) | The scores of all dimensions (reasonable diet, regular exercise, compliance with doctor's advice, blood glucose monitoring, foot care, prevention of high and low blood glucose) in the intervention group were significantly higher than those in the control group (*p*< 0.01). |
| Qin 2020[28] | China | One | Patients with DF with Wagner grade 0 | 67/67 | 53.19(7.85) | 53.84(8.02) | 39 (58.2) | 41 (61.2) | Transitional care based on WeChat platform (WeChat group, educational knowledge in the form of text, pictures, cartoons, and video) | 6 m | Routine nursing and telephone follow-up | 6 m | Y | Diabetes self-management ability (Diabetes Self-management Behavior Scale) | Mean ± SD: 74. 75±7.45 VS. 54. 34±6.13 (*p*< 0.05) |
| Hu 2018[29] | China | One | Patients with DF with Wagner grade 0 | 50/47 | 56.16(10.24) | 58.34(8.97) | 31 (62.0) | 29 (61.7) | Routine education + telephone follow-up education + platform management | 6 m | Routine education + telephone follow-up education | 6 m | N | Diabetes self-management ability (SDSCA) | Mean ± SD: 32.19±4.31 VS. 28.14±6.12 (*p*<0.001) |
| Bai 2022[30] | China | One | Patients with DF with Wagner grade 0-2 | 67/67 | 63.94(9.03) | 63.07(8.52) | 41 (61.2) | 37 (55.2) | Hospital-community integrated management of hierarchical medical system by medical alliance co-management | 6 m | Routine management | 6 m | Y | Foot care behaviors (DFSQ-UMA) | The scores of all dimensions (personal care, foot care, and wearing shoes and socks) in the intervention group were significantly higher than those in the control group (*p*< 0.01). |
| Wang 2023[31] | China | One | Patients with DF with Wagner grade 0-2 | 64/64 | 63.07(9.12) | 62.12(9.05) | 36 (56.3) | 34 (53.1) | Medical alliances co-manage hierarchical medical system (Medical alliance information platform, remote consultation) | 6 m | Routine nursing | 6 m | Y | Foot care behaviors (DFSQ-UMA) | The scores of all dimensions (personal care, foot care, and wearing shoes and socks) in the intervention group were significantly higher than those in the control group (*p*< 0.01). |

^a^This study applied the Wagner grading system to classify patients with diabetic foot, which is currently the most widely used grading system in clinical practice and research. Grade 0 indicates risk factors for foot ulcers but currently no ulcers are present, while grades 1 and above indicate varying degrees of ulcers or necrosis. A higher grade indicates a greater severity of the disease. The corresponding grade was extracted from the original literature, or graded according to its description (the grade in parentheses indicates our grading according to the description), if the original literature had neither grade nor sufficient description to judge, the grade was not recorded. ^b^ For non-ITT analysis studies, the sample size was the number of participants actually analyzed.

I: intervention group; C: control group; ITT: Intention-to-treat; DFU: diabetic foot ulcer; T2DM: type 2 diabetes mellitus; A1C: glycated hemoglobin; DF: Diabetic Foot; m: month; w: week; Y: Yes; N: No; DFCB: diabetic foot care behavior; QAD: Diabetes Self-care Activities Questionnaire; DFSBS: Diabetic Foot Selfcare Behavior Scale; SDSCA: Summary of Diabetes Self-Care Activities; 2-DSCS: Type 2 Diabetes Self-care Scale; DFSQ-UMA: diabetic foot self-care questionnaire of the University of Malaga; LSM: least squares mean; SE: standard error; 95% CI: 95% confidence interval
