# Supplementary figures and images for: Effects of Digital Intelligent Interventions on Self-Management of Patients With Diabetic Foot: Systematic Review
Source: J Med Internet Res. 2025 Mar 25;27:e64400. doi: 10.2196/64400 (PMC11979535; doi:10.2196/64400)

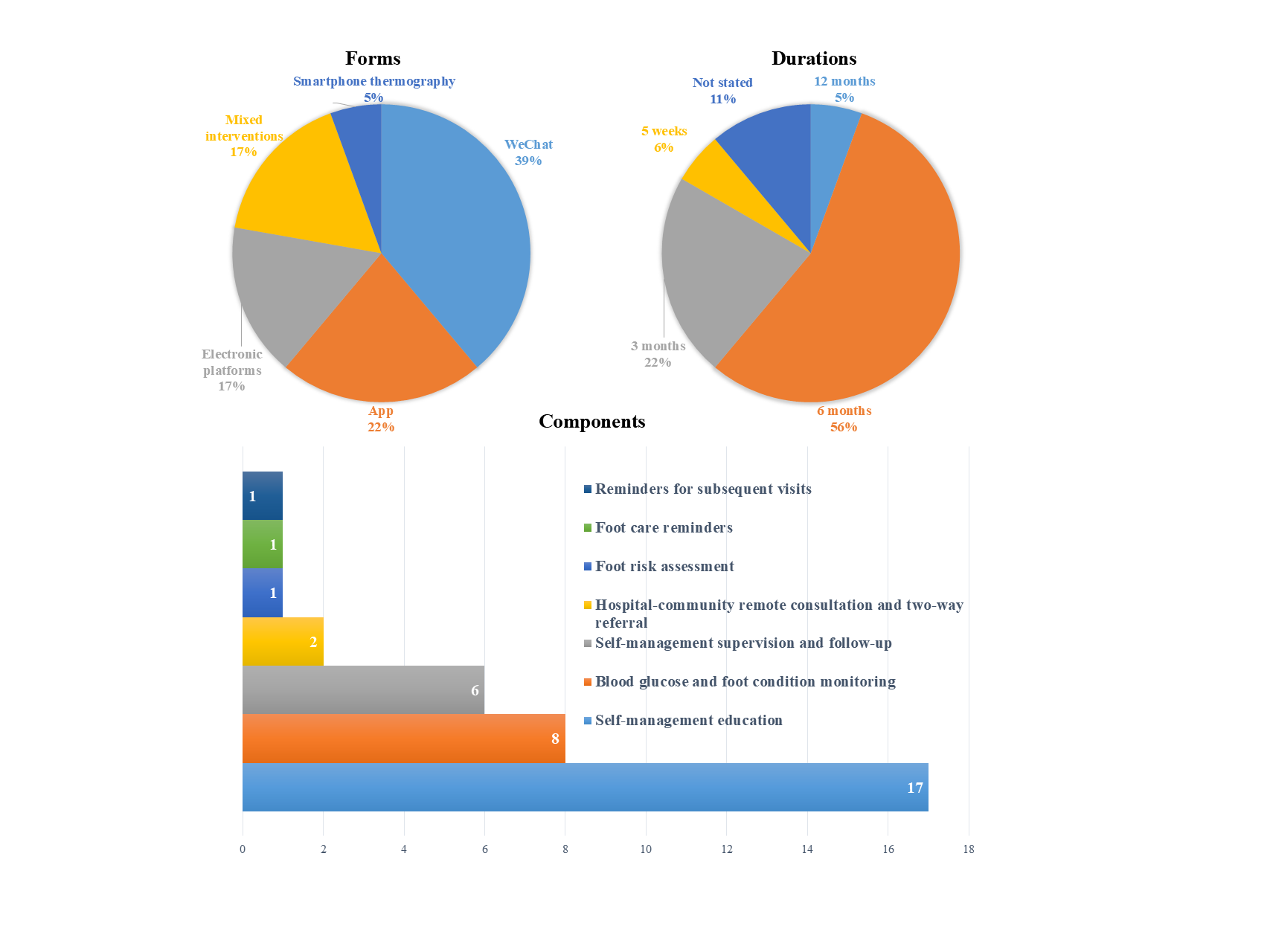

Supplement: Multimedia Appendix 3 [file jmir_v27i1e64400_app3.png]
